# Transcriptomic and Lipidomic Characteristics of Subcutaneous Fat Deposition in Small-Sized Meat Ducks

**DOI:** 10.3390/metabo15030158

**Published:** 2025-02-26

**Authors:** Hao Zheng, Cui Wang, Ao Zhou, Xing Chen

**Affiliations:** 1 Laboratory of Genetic Breeding, Reproduction and Precision Livestock Farming, School of Animal Science and Nutritional Engineering, Wuhan Polytechnic University, Wuhan 430023, China; 15926744416@163.com; 2 Institute of Animal Husbandry and Veterinary Science, Shanghai Academy of Agricultural Sciences, Shanghai 201106, China; cuiwang518@saas.sh.cn; 3 Institute of Animal Husbandry and Veterinary, Wuhan Academy of Agricultural Science, Wuhan 430345, China

**Keywords:** meat duck, subcutaneous fat deposition, lipidomics, transcriptomics, gene expression, adipocytokine signaling pathway

## Abstract

**Background:** Subcutaneous fat deposition is associated with ducks’ meat quality and the methods used to cook them. However, the reasons underlying the differences in the lipid deposition of small-sized Wuqin10 meat ducks remain unclear. **Method:** In the present study, to elucidate the metabolic mechanisms of lipid deposition, we comprehensively analyzed the transcriptomics and lipidomics of subcutaneous fat in Wuqin10 meat ducks with different subcutaneous thicknesses with six replicates. **Results:** A total of 1120 lipids were detected in the lipidomic analysis, and 39 lipids were inexorably regulated in the ducks with the thick subcutaneous layer compared to those with the thin layer; further, the up-regulated lipids were primarily triglycerides (TGs), which may have resulted in adipocyte enlargement. Furthermore, the transcriptomic analysis identified 265 differentially expressed genes (DEGs), including 119 down-regulated and 146 up-regulated genes. Gene Ontology (GO) and Kyoto Encyclopedia of Genes and Genomes (KEGG) analyses showed that the DEGs were significantly enriched in the histidine, arginine, proline metabolism signaling and adipocytokine signaling pathways. The protein–protein interaction (PPI) network in Cytoscape 3.8.2 identified hub genes HSP90AA1, RUNX2, ACTN2, ACTA1, IL10, CXCR4, EGF, SOCS3 and PTK2, which were associated with the JAK-STAT signaling pathway and regulation of adipocyte hypertrophy. **Conclusion:** Taken together, our findings reveal the patterns of lipids and the gene expression of subcutaneous fat, providing a basis for future studies of subcutaneous fat deposition in small-sized meat ducks.

## 1. Introduction

Ducks are economically important domestic waterfowl, and annual duck meat production is increasing globally owing to its high nutritional value and content of essential unsaturated fatty acids [1]. Different ducks breeds have different processing methods; for example, large-sized meat ducks, such as Peking ducks with high fat deposition, are suitable for roasting, whereas small-sized meat ducks with higher meat quality can be used for braising [2]. To date, excessive fat deposition has always hindered the development of meat ducks, and most research about subcutaneous fat deposition has focused on large-sized meat ducks; therefore, excessive fat deposition in small-sized meat ducks also needs to be resolved. Subcutaneous fat is the largest adipose tissue and one of the main factors affecting the growth, meat quality, feed conversion rate and processing method of ducks [3], and excessive subcutaneous fat deposition can result in an increase in inflammation and feeding cost. Therefore, understanding the mechanism underlying subcutaneous fat deposition in small-sized meat ducks is critical for improving meat quality. Previous studies have shown that subcutaneous fat deposition is a complex trait resulting from pre-adipocyte differentiation and maturation, lipid transport and accumulation, and adipocyte hypertrophy [4,5,6]. Pre-adipocyte differentiation and adipogenesis affect the number of fat cells and enlargement of adipocytes, and these processes are characterized by gene expression [7,8]. The CCAAT/enhancer-binding protein (C/EBP) family [9], peroxisome proliferator-activated receptor (PPAR) [10], fatty acid-binding protein (FABP4) [11], lipoprotein-lipase (LPL) [12] and fatty acid synthase (FAS) [13] are known to play important roles in subcutaneous fat deposition. PPARγ is key in inducting fatty acid synthesis, whereas FABP4 regulates PPARγ expression to affect fatty acid transportation and metabolism [14]. The early stages of adipogenesis can be blocked by Wnt signaling family members, such as β-catenin Wnt10b and Dkk1, resulting in undifferentiated preadipocyte accumulation with a decrease in PPARγ and C/EBPα expression [15], indicating the importance of comparing the molecular mechanisms of subcutaneous fat deposition in different duck strains and screening the related candidate genes to speed up the improvement of subcutaneous traits in ducks.

Genetic selection has significantly improved subcutaneous fat deposition in ducks [16]; further, some candidate genes and molecular markers related to subcutaneous fat deposition have been identified [17,18,19]. However, the genetic regulation mechanisms and related signaling pathways remain poorly understood. Transcriptome sequencing (RNA-seq) is a powerful tool that allows the exploration of gene and transcript expression at a global level [20]. Using transcriptome analysis, SNCG, PLPPR4 and VAMP1 genes were found to be involved in the growth and metabolism of breast muscles of Jinghai yellow chickens with fast and slow growth [21]. Further, several differentially expressed genes in subcutaneous adipose tissue have been identified during the development of Muscovy ducks [22].

In this study, we aimed to identify differentially expressed genes (DEGs) in the subcutaneous fat tissue deposition of two different lines of small-sized meat ducks using RNA-seq and bioinformatics and to analyze their functional roles. These results can provide more knowledge on the mechanism of subcutaneous fat deposition and directional breeding in ducks.

## 2. Materials and Methods

### 2.1. Experimental Materials

Wuqin10 meat ducks were selected from a cross between Peking ducks and Liancheng white ducks (a local breed in Fujian, China) according to subcutaneous fat thickness (3 mm) via several generations, and were raised to ten weeks of age on the same farm under the same standard management conditions. Twelve female Wuqin10 meat ducks with different subcutaneous fat deposition (thin line and thick line) were used in this experiment (Supplementary Table S1), and were fed a commercial corn–soybean-based diet ad libitum with free access to water. All ducks were healthy and had not been treated with antibiotics. They were kept together from the duckling to slaughter stages, and fasted for 8 h before being slaughtered by electric shock and cervical dislocation according to the national and institutional guidelines for the ethical conduct and treatment of experimental animals. Then, chest subcutaneous fat tissue samples were collected and immediately stored in liquid nitrogen and 4% paraformaldehyde for the following experiments.

### 2.2. Histological Analysis

The subcutaneous fat tissues of small-sized meat ducks were 4% paraformaldehyde-fixed for 24 h at room temperature, and then were embedded in paraffin, and 5 μm thick serial sections were made. The sections were then stained with hematoxylin and eosin following the standard protocols. Microscopic observations were made and photographs were taken with a Nikon 90i microscope (Nikon, Japan). At least 3 fields of view per section were selected (×200 magnification). The average surface area of adipocytes and the average adipocyte number in each section were calculated using Image-pro plus 6.0. SPSS software 29 was used to compare the significance of repeated measurements between the two duck strains, with a significance criterion of *p* < 0.05.

### 2.3. Lipid Sample Preparation and Lipidomic Analysis

The lipid extraction of subcutaneous fat samples was performed as follows: a total of 700 μL of precooled chloroform/methanol (2:1 *v*/*v*) was added into a 20 mg sample to dissolve the lipid residue and homogenized by a superfine homogenizer; the samples were then vortexed for 2 min and incubated for 15 min at 4 °C. After centrifuging at 3000 rpm for 15 min at 4 °C, the lower chloroform layers were collected and washed twice. Lipid detection was performed using an UHPLC-MS/MS-based targeted metabolomics platform. A quality control (QC) sample prepared by combining the same volume of all experimental samples was used to monitor system stability. The raw data were processed using Compound Discoverer 3.01 (CD3.1, Thermo Fisher, Waltham, MA, USA) to perform peak alignment, peak picking, and quantitation for each metabolite following normalization to the total spectral intensity. The supervised Orthogonal Partial Least Squares Discriminant Analysis (OPLS-DA) model was used to mine for different metabolites. After the univariate analysis (*t*-test), the significantly differential lipids were identified as follows: variable importance in the projection (VIP) value > 1, |log_2_fold-change| > 1, and *p*-value < 0.05.

### 2.4. RNA-Seq and Differentially Expressed Gene (DEG) Analysis

Total RNA was extracted from subcutaneous fat samples using TRIzol Reagent according to the manufacturer’s instructions. The integrity of the obtained RNA was evaluated using an Agilent Bioanalyzer 2100 system, and RNA sequencing was carried out on an Illumina HiSeq platform. After evaluating, trimming and filtering the raw data, high-quality clean reads were obtained and mapped to the duck genome (CAUduck1.0) using HISAT2 software (version 2.2.1). HTSeq2.0 was used as the original expression level of the gene, and the R package DEGseq2 was used to extract the read count value and gene-level expression value for identifying differentially expressed genes (DEGs) following the criteria |log_2_FoldChange) | ≥ 1 and *p* < 0.05.

### 2.5. qPCR Validation

To verify the transcriptomic analysis results, we performed qPCR. After extracting total RNA of subcutaneous fat, the first-stand cDNA was synthesized as following the manufacturer’s instructions. The relative expression level of candidate genes was detected on an Archimed R4 PCR instrument (ROCGENE, Beijing, China) using the 2^−ΔΔCT^ method. GAPDH was selected as the internal control gene for the normalization of target genes. The experiments were performed with at least three biological replicates and three technical replicates per biological replicate. The information about the primers is listed in Supplementary Table S2.

### 2.6. Functional Enrichment Analysis and Protein–Protein Interaction Network Analysis

Functional enrichment analysis of the DEGs was performed using the Gene Ontology (GO) [23] and Kyoto Encyclopedia of Genes and Genomes (KEGG) pathways (https://www.genome.jp/kegg/, accessed on 24 April 2024) [24] with the R package clusterProfiler, with *p* < 0.05 as the threshold value for screening significantly enriched GO terms and pathways. The protein–protein interaction (PPI) of the DEGs was analyzed using the Search Tool for the Retrieval of Interacting Genes (STRING) database, and then, the hub genes were identified with the maximal clique centrality (MCC) method in the cytoHubba plugin app in Cytoscape 3.8.2.

## 3. Results

### 3.1. Adipocyte Morphology of Subcutaneous Fat in Ducks

**Figure 1:**
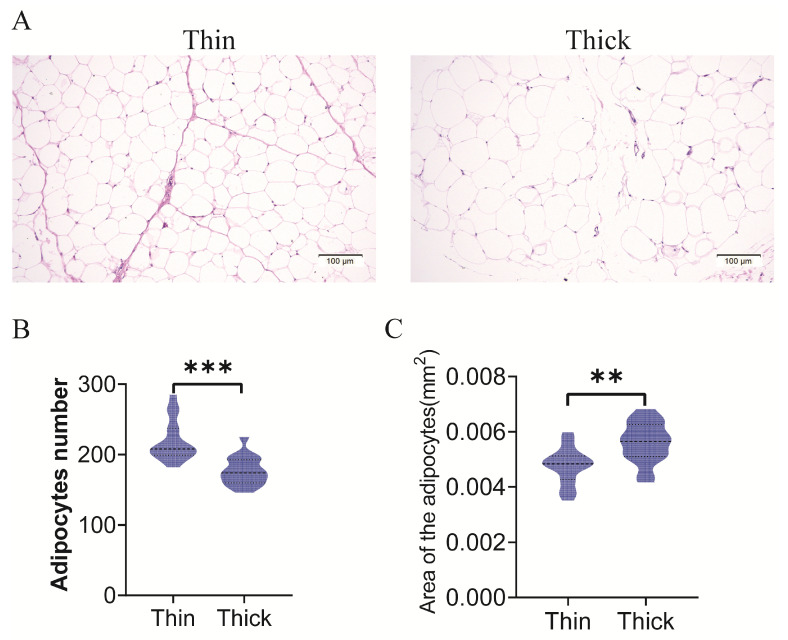
Comparison of subcutaneous adipose cells between two strains of small-sized meat ducks. (**A**) Representative H&E staining pictures of subcutaneous adipose cells of two strains of small-sized meat ducks (thin and thick). (**B**,**C**) Adipocyte number and area were compared in two strains of small-sized meat ducks. **: *p* < 0.01; ***: *p* < 0.001.

Fat formation can result from hypertrophy and hyperplasia [25]. In this study, Wuqin10 meat ducks with different subcutaneous fat thicknesses (thin and thick groups) were selected (Table 1). Histological analyses of the subcutaneous fat revealed that the adipocytes of the ducks with thick subcutaneous fat appeared larger than those in the thin ducks (Figure 1A). After calculating the number and area of the adipocytes in the thick and thin groups, we found that ducks with thick subcutaneous fat had fewer adipocytes and a larger adipocyte area than thin ducks (Figure 1B,C), suggesting that the difference in subcutaneous fat deposition among individuals of the same breed may be caused by adipocyte hypertrophy and hyperplasia.

### 3.2. Comparative Analysis of Lipid Metabolism Characteristics

**Figure 2:**
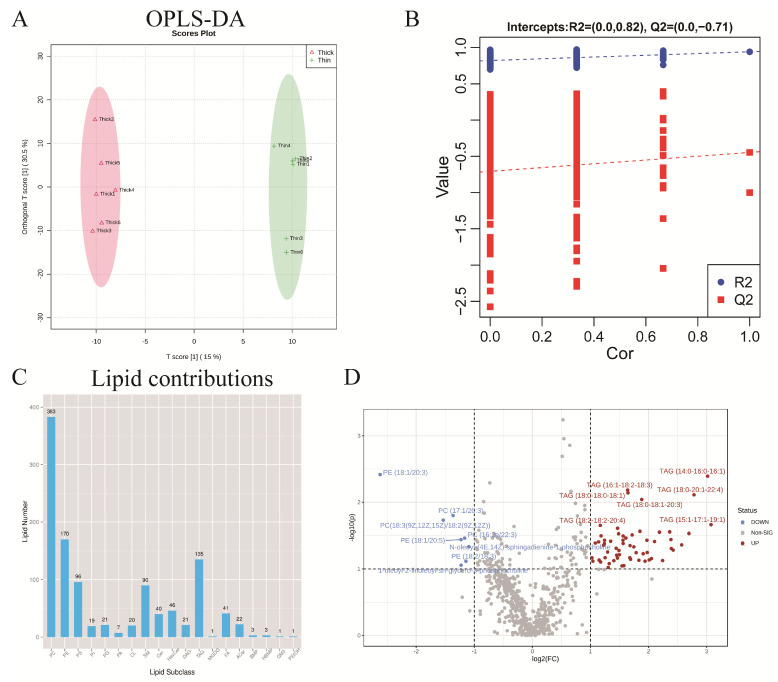
Lipid composition and changes detected in two strains of small-sized meat ducks. (**A**) Orthogonal partial least squares discriminant analysis (OPLS-DA) of subcutaneous adipose tissue from two strains of small-sized meat ducks (thin and thick); X axes represent predictive component, and Y axes represent orthogonal component. (**B**) Model validation diagram of OPLS-DA. R2 represents explained variance of model, and Q2 indicates predictive ability of model. (**C**) Lipid contributions. (**D**) Volcano graph of differential lipid molecules in two strains of small-sized meat ducks.

Adipocyte hypertrophy and hyperplasia are associated with lipid metabolism [26]. To determine the changes in overall lipid composition and distribution between the thick and thin groups, mass spectrometry-based lipidomic analysis was applied. Orthogonal partial least squares discriminant analysis (OPLS-DA) plots showed that the two different groups of subcutaneous fat (thin and thick) were well separated (Figure 2A); further, the model validation diagram for OPLS-DA showed no overfitting of this model (Figure 2B), indicating good repeatability and stability of the samples. Based on the lipidomics results, 1120 lipid molecules were identified (Supplementary Material Data 1), and the types and amounts of lipid metabolites are listed in Figure 2C. Compared with the thin group, the thick group had 33 up-regulated lipids, primarily comprising triglycerides (TGs), while only six lipid metabolites were down-regulated, including phosphatidylcholine (PC) and phosphatidylethanolamine (PE) (Figure 2D).

### 3.3. Transcriptomics Profiles of Subcutaneous Fat in Ducks

To analyze the gene expression patterns between the thin and thick groups of subcutaneous fat in ducks, raw data from RNA-seq were cleaned by removing the adaptor and low-quality sequences to obtain 6 Gb clean reads. The percentage of Q30 (clean data mass of no less than 30 bases) was higher than 93.5%, and an average of 80% of the clean reads were aligned with the reference genome, indicating that the sequencing data could be used for subsequent analysis (Table 2).

**Figure 3:**
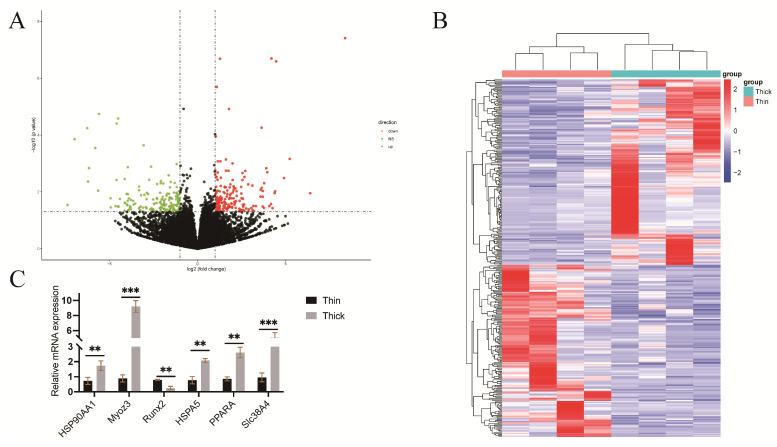
Differentially expressed genes (DEGs) of subcutaneous fat in the thick strain of meat ducks compared with the thin strain of small-sized meat ducks. (**A**) The volcano plot shows the differentially expressed genes (DEGs). The red dots represent significantly up-regulated genes, and the green dots represent significantly down-regulated genes. (**B**) Hierarchical clustering analysis was performed based on DEGs in the two strains of small-sized meat ducks. (**C**) Validation of 6 DEGs between two different duck lines with different thicknesses. **: *p* < 0.01; ***: *p* < 0.001.

Compared with the thin group, a total of 265 DEGs were screened based on *p* ≤ 0.05 and |log_2_FoldChange| ≥ 1, including 146 up-regulated and 119 down-regulated genes (Figure 3A). Hierarchical clustering analysis showed the same cluster as the sample group, but differences between groups, suggesting obvious differences between the different types of lipid depositions in the subcutaneous adipose tissue of ducks (Figure 3B). Further, to verify the transcriptomic analysis results, we determined the expression level of some DEGs related to lipid biosynthesis using qPCR. The qPCR results were in agreement with the RNA-seq results (Figure 3C).

### 3.4. Functional Enrichment Analysis of DEGs

**Figure 4:**
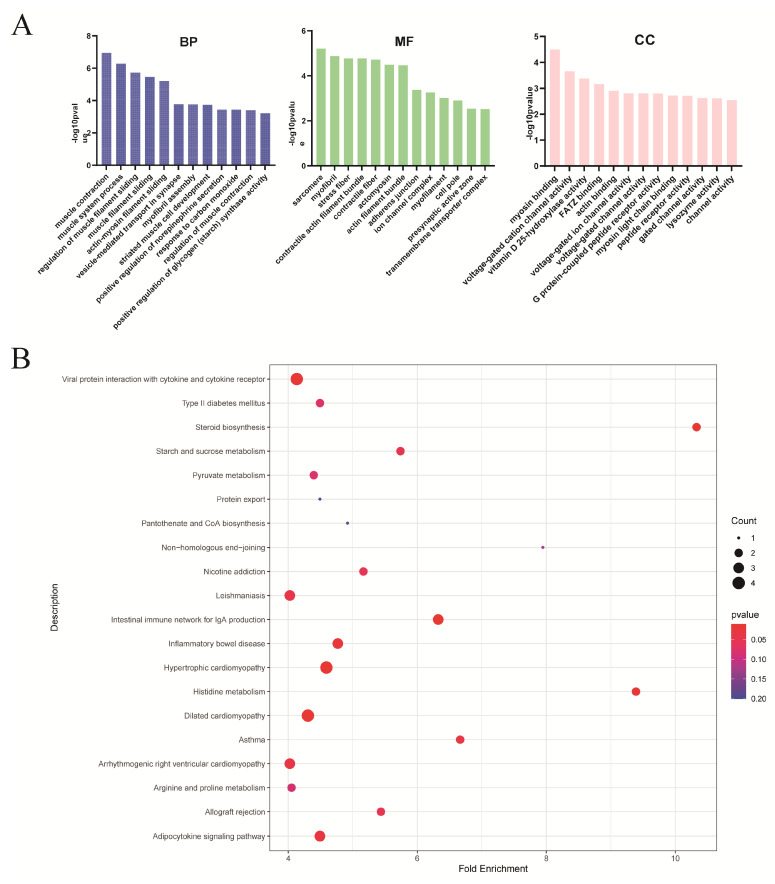
The GO terms and KEGG enrichment analysis of differentially expressed genes (DEGs) in the two strains of small-sized meat ducks. (**A**) The significant biological processes, molecular functions and cellular components of the DEGs are shown. (**B**) KEGG pathway enrichment analysis of the DEGs with the significant pathways. The size of the dots indicates the number of genes enriched in the pathway, and the color of the dots represents the p-value of the pathway.

To further elucidate the functional roles of the DEGs, Gene Ontology (GO) functional enrichment was analyzed, and the results showed that the biological processes were mainly enriched in muscle contraction and muscle system processes, whereas cell components were mainly enriched in myosin binding, channel activity and actin activity (Figure 4A). RUNX2, Myoz3, SLC38A4 and HSPA5 were involved in lipid synthesis and inflammatory responses. Further enrichment analysis of the Kyoto Encyclopedia of Genes and Genomes (KEGG) pathway was conducted, and the results showed that the DEGs were significantly enriched in steroid biosynthesis; the adipocytokine signaling pathway; and metabolism, including histidine, arginine and proline metabolism (Figure 4B). Further, some DEGs were enriched in cytokine–cytokine receptor pairs related to inflammation.

### 3.5. Protein–Protein Interaction (PPI) Network Analysis of DEGs

**Figure 5:**
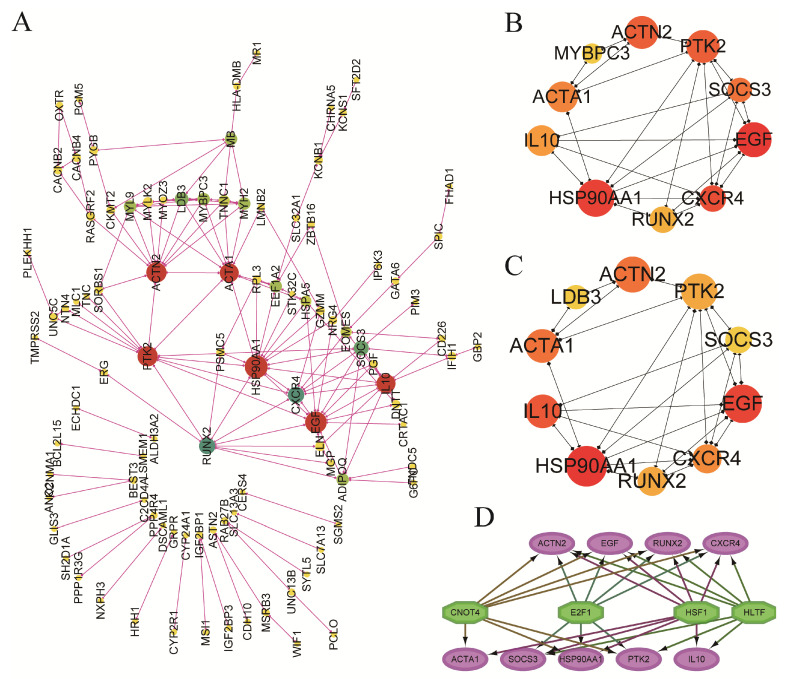
The protein–protein interaction (PPI) network analysis of DEGs and the identification of hub genes. (**A**) The PPI network analysis of differentially expressed genes (DEGs) in the two strains of small-sized meat ducks. (**B**,**C**) The MCC and MNC algorithms of the Cytohubba plugin to obtain the hub genes. (**D**) The transcription factors (TFs) of the hub genes were identified by using the iRegulon plugin in Cytoscape.

To explore the interaction of the obtained DEGs and acquire the hub genes related to adipocyte hypertrophy and hyperplasia, the corresponding PPI network was constructed using the STRING online database and visualized using Cytoscape software (Figure 5A). The cytoHubba plugin was then used to identify hub genes based on the maximum neighborhood component (MCC) and maximum neighborhood component (MNC) methods; nine hub genes, including ACTN2, ACTA1, IL10, HSP90AA1, RUNX2, CXCR4, EGF, SOCS3 and PTK2, were obtained (Figure 5B,C and Table 3), and they were related to the positive regulation of metabolic processes, tissue morphogenesis, cell differentiation and the regulation of the actin cytoskeleton. Transcription factors (TFs) are known to directly interact with their target genes to specifically regulate gene expression. Here, we used the iRegulon plugin and found that these nine hub genes were regulated by one of four TFs (HSF1, HLTF, E2F1 or CNOT4) (Figure 5D).

## 4. Discussion

Subcutaneous fat deposition varies greatly among duck breeds. Meat ducks deposit more subcutaneous fat than laying ducks. The two lines used in this study were derived from a hybrid population of meat and laying ducks. These two lines have large differences in subcutaneous fat deposition, but similar body sizes and growth rates, reducing the genetic background interference of other traits and making them a good model for studying sebaceous deposition. Histological analyses showed that the adipocytes of ducks with the thick subcutaneous fat were larger than those of the ducks with the thin layer. This increase in subcutaneous fat tissue mass was attributed to adipocyte hyperplasia and hypertrophy. Further, the recruitment of multipotent stem cells to adipose tissue to produce new preadipocytes, as well as the mitotic clonal expansion of existing preadipocytes, contribute to hyperplasia. Increased lipogenesis and storage of triacylglycerol led to hypertrophy of mature adipocytes.

The lipidomic analysis indicated that fat deposition was associated with the metabolism of triglycerides [27,28]. We found that TGs were significantly higher, whereas phosphatidylcholine (PC) and phosphatidylethanolamine (PE) contents were decreased, in the ducks with thick subcutaneous fat. Previous studies have shown that the metabolism of TG, PC, and PE is affected by transcriptional alteration [29]. However, combined transcriptomics and lipidomic analyses did not reveal significant pathways related to lipid metabolism, indicating, like in other avian species, that the deposited lipid in the duck subcutaneous fatty tissue was not locally synthesized [30].

Transcriptome analysis identified DEGs that were enriched in muscle contraction and muscle system processes, steroid biosynthesis, the adipocytokine signaling pathway and metabolism-related pathways. ZDHHC22, one of the top five DEGs, is a palmitoyltransferase that can regulate protein palmitoylation; previous studies have shown that some adipose proteins (GLUT4, ARL15, CD63) are palmitoylated in subcutaneous adipose tissue [31,32,33], suggesting that lipid metabolism and adipocyte differentiation can be regulated by these key DEGs to affect subcutaneous fat deposition. PPI network analysis indicated that LDB3 is related to a large number of genes. LDB3 is expressed in subcutaneous adipose tissue and plays an important role in cardiometabolic phenotypes. The LDB3 promoter responds to lipid uptake in human adipocytes, and the GxE SNP rs10788522 regulates adipocyte expression [34]. Further, LDB3 can bind non-muscle-specific alpha-actinin isoforms to maintain proper tissue functions [35]. Similarly, the level of PCDH7 mRNA expression was significantly increased in the duck line with thick subcutaneous fat. A genome-wide scan for interactions between SNP markers and traditional epidemiological risk factors identified a genome-wide significant locus, rs6448771, from the PCDH7 gene, which was an adipose tissue-specific eQTL for PCDH7 [36].

RUNX2 is a key regulator of mesenchymal cell differentiation to osteoblasts. RUNX2 deficiency leads to the differentiation of mesenchymal cells into adipocytes, and the dephosphorylation of RUNX2 supports adipogenesis [37]. RUNX2 silencing causes an increase in adipogenic markers and fat droplets by reducing the expression of DLK1 [38]. A genetic polymorphism in the RUNX2 promoter is associated with triglyceride (TG) levels [39]. The results of our study showed a decrease in RUNX2 expression, suggesting that RUNX2 may play a negative role in subcutaneous fat accumulation. Further, significant DEGs were related to steroid biosynthesis, amino acid metabolism, and the adipocytokine signaling pathway. Taken together, the key DEGs identified in this study may be important candidate marker genes for subcutaneous fat deposition.

Hub gene analysis revealed the involvement of a heat shock protein HSP90AA1, belonging to the HSP90 family. Owing to their overall covering of feathers and their lack of sweat glands, ducks are susceptible to heat stress. Previous studies have shown that fat deposition is increased under heat stress [40], and that heat exposure improves lipid metabolism and regulates plasma triglyceride absorption and storage in adipose tissue [41]. HSP90AA1 regulates autophagy, which controls adipose mass and adipogenesis [42,43]. HSP90AA1 is also a lipid droplet (LD)-associated protein, and HSP90 interacts with PPARγ to regulate adipocyte differentiation, while HSP90 inhibition (geldanamycin) inhibits adipocyte differentiation and fat mass accumulation [44,45]. HSP90 interference reduces the mRNA and protein expression of PPARγ, FAS and SREBP-1c, which are associated with adipocyte differentiation to regulate the accumulation of triglyceride [46]. Notably, lower expression of HSP90AA1 showed better adapted genotypes in Indian sheep, and single-nucleotide polymorphisms (SNPs) in HSP90 genes were found to affect triglyceride metabolism [47]. HSP90AA1 played an important role in atrial fibrillation by regulating lipid metabolism and biosynthesis [48]. Focal adhesion kinase 1 (FAK1, PTK2) is a non-receptor protein–tyrosine kinase related to reorganization of the actin cytoskeleton and cell proliferation [49]. During adipocyte differentiation, focal adhesion kinase is cleaved by calpain, and the inhibition of FAK activity increases the expression of adipogenic marker genes AP2 and LEP, resulting in lipid accumulation [50]. Further, inflammation in adipose tissue is well known to be associated with human obesity [51]. Excessive adipogenesis leads to the development of proinflammatory conditions, and inflammasome activation induces lipid deposition [52]. This study indicated the involvement of some inflammation-related genes (IL10, CXCR4 and SOCS3) in subcutaneous adipose tissue deposition. CXCR4 is highly expressed on the surface of native murine adipose stromal cells (ASCs) from adipose tissue (AT) [53]. The expression level of SOCS-3 is significantly higher in the adipose tissue of obese pigs than in lean pigs [54]. Taken together, inflammation-related genes play important roles in regulating subcutaneous fat deposition. However, further details need to be investigated in the future.

## 5. Conclusions

In summary, our lipidomic and transcriptomic analyses revealed that triglycerides were significantly higher in duck lines with thick subcutaneous fat, and 265 differentially expressed genes were associated with the metabolism and adipocytokine signaling pathways. Moreover, nine regulatory hub genes (ACTN2, ACTA1, IL10, HSP90AA1, RUNX2, CXCR4, EGF, SOCS3 and PTK2) were identified and may be associated with subcutaneous fat deposition. Overall, this study fills in the gaps in knowledge regarding the regulatory mechanisms of subcutaneous fat deposition and provides potential targets for genetic selection in ducks.

## Figures and Tables

**Table 1:** Carcass traits of quality meat ducks at 10 weeks.

Carcass Traits	Group Thick	Group Thin
body weight/g	2067 ± 83.6	2013 ± 89.4
slaughter weight/g	1848 ± 85.6	1826 ± 100.2
slaughter rate/%	88.2 ± 2.0	89.1 ± 1.1
semi-eviscerated rate/%	81.1 ± 2.0	81.9 ± 3.28
eviscerated rate/%	76.7 ± 1.9	77.3 ± 1.3
breast rate/%	13.9 ± 0.8	14.0 ± 1.1
leg muscle rate/%	9.6 ± 0.7	9.5 ± 0.9
Sebum rate/%	31.6 ± 2.8	28.0 ± 2.9
sebum thickness/mm	3.2 ± 0.1	2.1 ± 0.1

**Table 2:** Summary of sequence quality and alignment information.

Sample Name	Raw Reads	Q30 (%)	Clean Reads	Clean Bases	Unique Mapped Reads	Mapped Ratio (%)
Thin-1	47126982	94.18	44368926	6.66G	35344941	79.66%
Thin-2	41991798	93.98	39701218	5.96G	31860484	80.25%
Thin-3	42757478	93.82	39994494	6.0G	31651576	79.14%
Thin-4	43516768	94.16	40708084	6.11G	32488770	79.81%
Thick-1	43203534	93.57	40815532	6.12G	32120760	78.7%
Thick-2	48121678	93.86	45230024	6.78G	35948497	79.48%
Thick-3	49407598	93.9	45230024	6.89G	37060375	80.66%
Thick-4	45396384	94.05	42184968	6.33G	32891587	77.97%

**Table 3:** hub genes expression.

Gene_Name	Log_2_FoldChange	*p* Value
PTK2	−3.24	0.044
RUNX2	−1.38	0.039
CXCR4	1.11	0.021
SOCS3	1.14	0.042
EGF	2.17	0.036
ACTN2	2. 44	0.0038
ACTA1	2.71	0.0018
IL10	3.64	5.51 × 10^−5^
HSP90AA1	8.38	3.87 × 10^−8^

## Data Availability

The original contributions presented in this study are included in the article and Supplementary Material. Further inquiries can be directed to the corresponding authors.

## Supplementary Materials

The following supporting information can be downloaded at: https://www.mdpi.com/article/10.3390/metabo15030158/s1. Table S1: The information of samples from two hybrid small-meat duck strains; Table S2: Fluorescence quantitative primer information; Supplementary Data 1: Lipidomic results.
