# Supplementary material for: Transcriptomic and Lipidomic Characteristics of Subcutaneous Fat Deposition in Small-Sized Meat Ducks
Source: Metabolites. 2025 Feb 26;15(3):158. doi: 10.3390/metabo15030158 (PMC11944229; doi:10.3390/metabo15030158)
Supplement: Supplementary file 1 [file metabolites-15-00158-s001.zip › Supplementary table.docx]

Supplementary Table S1 The information of samples from two hybrid small-meat duck strains

| Samples | Body weight(g) | Subcutaneous fat thickness (mm） |
| --- | --- | --- |
| Thin-1 | 2076 | 2.1 |
| Thin-2 | 2054 | 2.1 |
| Thin-3 | 2023 | 2.0 |
| Thin-4 | 2008 | 2.0 |
| Thin-5 | 2134 | 2.2 |
| Thin-6 | 2074 | 2.2 |
| Thick-1 | 2007 | 3.2 |
| Thick-2 | 2126 | 3.3 |
| Thick-3 | 2103 | 3.1 |
| Thick-4 | 2047 | 3.0 |
| Thick-5 | 2129 | 3.0 |
| Thick-6 | 1995 | 3.0 |

Supplamenty table S2 Fluorescence quantitative primer information

| Genes | Primer sequence (5’-3’) |
| --- | --- |
| GAPDH | F:GGAGCTGCCCAGAACATTATC  R:GCAGGTCAGGTCCACGACA |
| HSP90AA1 | F:GCCAGTTTGGTGTCGGTTTC  R:GCTGACGACTCCCAAGCATA |
| MYOZ3 | F:AGGTGCCCAAGAAATCGGAG  R:TGACGTTGAACTTCTCCGGG |
| RUNX2 | F:TTAGGGCGCATTCCTCATCC  R:GGACTTGGTGCAGAGTTCAGA |
| HSPA5 | F:AATGGCCGGGTGGAAATCAT  R:TGGACGTCAGCTGGTTCTTG |
| PPARA | F:AATGGCTGACCTCCGACAAC  R:ACTTGTCCCTGTAGATTTCCTG |
| ZDHHC22 | F:CCTCTCAGCTAATGCCCTGG  R:GCCCAAGTTTAAGCCCTTGC |
